# Supplementary material for: A Likelihood Approach for Real-Time Calibration of Stochastic Compartmental Epidemic Models
Source: PLoS Comput Biol. 2017 Jan 17;13(1):e1005257. doi: 10.1371/journal.pcbi.1005257 (PMC5240920; doi:10.1371/journal.pcbi.1005257)
Supplement: S1 File — (TAR.GZ) [file pcbi.1005257.s014.tar.gz › HSPH_Online-SI-Revision/EnKF07/n10-severe/n10-severe_EnKF_table8.pdf]

1	R0	{1.68952, 11.8402}	{2.43651, 7.12159}	{{1.71961, 3.31443}, {3., 12.7105}}	{44.213, 41.9105}	inf	198.	1001.68	{298.373, 1952.64}	421.311	0.686839	newly	99.	1457.22	{304., 3899.}	1386.06	0.990139	Reff	0.571396	0.996036	{0.559435, 1.8168}	76.4533	ar	6872.	17432.6	{15069., 24526.}	153.676
2	R0	{1.71739, 9.62661}	{2.66539, 7.35506}	{{2.09565, 0.175054}, {3., 12.7206}}	{55.2, 51.1026}	inf	184.	204.8	{7.83783, 128.685}	114.639	0.59106	newly	116.	452.82	{6., 449.}	376.931	0.627024	Reff	0.569658	0.726072	{0.442314, 1.37267}	45.4666	ar	6874.	15346.8	{14105., 17705.}	123.258
3	R0	{1.48936, 6.23975}	{2.37001, 3.88355}	{{1.46315, 1.45834}, {3., 9.37044}}	{60.5563, 59.8374}	inf	38.	578.115	{52.1161, 299.372}	1430.2	0.784841	newly	40.	543.14	{68., 1371.}	1265.3	0.847727	Reff	0.649957	0.398636	{0.0426605, 0.641836}	57.7154	ar	5666.	15291.3	{14099., 20168.}	169.879
4	R0	{1.62394, 7.51135}	{1.73141, 9.72475}	{{0.035223, 6.89205}, {3., 12.1532}}	{89.9809, 36.5579}	inf	86.	1582.33	{43.9746, 7253.81}	1760.46	0.93926	newly	66.	802.58	{21., 3134.}	1146.09	0.896892	Reff	0.601507	0.218865	{0.00822294, 0.532522}	64.6717	ar	6417.	17289.6	{15101., 25587.}	169.434
5	R0	{1.53632, 8.57652}	{1.94924, 1.87701}	{{0.230572, 0.981084}, {3., 3.66497}}	{91.2598, 78.1145}	inf	148.	497.153	{72.4377, 340.635}	253.575	0.424643	newly	112.	662.93	{111., 805.}	498.241	0.611479	Reff	0.610687	0.402819	{0.0521868, 0.646387}	44.547	ar	6260.	14460.9	{13498., 15246.}	131.005
6	R0	{1.47166, 5.7434}	{2.44047, 4.87633}	{{1.51418, 3.03281}, {3., 6.29092}}	{65.8308, 25.5684}	inf	52.	221.322	{10.302, 622.966}	344.746	0.60188	newly	51.	305.12	{8., 896.}	517.922	0.675921	Reff	0.657979	0.648809	{0.0817547, 1.51565}	51.575	ar	5650.	15446.7	{13670., 21261.}	173.392
7	R0	{1.48179, 8.87369}	{2.0694, 3.19842}	{{0.797363, 0.369937}, {3., 6.22527}}	{68.0835, 63.9561}	inf	96.	429.122	{54.3619, 542.949}	374.097	0.341764	newly	76.	508.91	{42., 1070.}	592.645	0.474185	Reff	0.721187	0.337625	{0.0674956, 0.738121}	58.3866	ar	5233.	16119.4	{14286., 20934.}	208.034
8	R0	{1.53669, 6.47807}	{1.80968, 6.67804}	{{0.454236, 3.63697}, {3., 10.1073}}	{83.1736, 49.5755}	inf	51.	940.781	{362.163, 1075.77}	1744.67	1.07889	newly	34.	895.09	{266., 1102.}	2532.62	1.15859	Reff	0.629736	0.344489	{0.067174, 0.73408}	53.2961	ar	5934.	17794.5	{16212., 21144.}	199.874
9	R0	{1.57227, 6.20989}	{2.36193, 11.5058}	{{1.40442, 2.86161}, {3., 19.8258}}	{58.7441, 137.586}	inf	66.	257.021	{9.28473, 302.474}	328.072	0.601007	newly	57.	290.51	{5., 543.}	455.596	0.658222	Reff	0.625135	0.694594	{0.0488016, 1.4046}	59.1893	ar	6136.	16368.2	{14652., 20345.}	166.757
10	R0	{1.54936, 6.50938}	{2.23455, 5.08191}	{{1.33421, 3.36428}, {3., 6.54508}}	{56.8943, 22.5217}	inf	32.	240.281	{80.683, 230.454}	652.456	0.542225	newly	31.	415.65	{55., 323.}	1241.71	0.569442	Reff	0.582559	0.501835	{0.228824, 0.704833}	43.3878	ar	6275.	16058.5	{14771., 19929.}	155.912
11	R0	{1.42179, 3.18601}	{1.91081, 5.26028}	{{0.626456, 1.0996}, {3., 12.07}}	{85.4873, 114.802}	inf	5.	695.921	{45.1125, 1679.87}	13818.4	1.7743	newly	7.	948.52	{26., 3847.}	13450.6	1.64265	Reff	0.665398	0.455411	{0.0721488, 0.790656}	38.9535	ar	5322.	16259.8	{14906., 19494.}	205.52
12	R0	{1.48035, 6.46183}	{2.27774, 2.98479}	{{0.591062, 2.41806}, {3., 3.70764}}	{89.8519, 53.809}	inf	58.	342.026	{36.9775, 259.014}	504.414	0.688851	newly	54.	446.21	{50., 487.}	738.574	0.790525	Reff	0.636995	0.39251	{0.0904421, 0.645953}	42.4506	ar	5793.	16248.2	{15200., 16476.}	180.48
13	R0	{1.75755, 8.53854}	{1.95565, 6.7576}	{{0.388848, 3.66101}, {3., 9.61598}}	{73.5591, 33.981}	inf	105.	1051.41	{374.562, 2208.95}	902.809	0.825295	newly	83.	984.2	{285., 2488.}	1087.25	0.821911	Reff	0.574719	0.426313	{0.0766304, 0.779447}	55.991	ar	6832.	18189.9	{16604., 21742.}	166.246
14	R0	{1.47355, 4.89451}	{2.71326, 11.0981}	{{2.07503, 8.2727}, {3., 12.1727}}	{84.1307, 126.746}	inf	18.	340.009	{32.1774, 303.452}	1793.79	0.932234	newly	20.	211.43	{16., 222.}	968.55	0.673702	Reff	0.635542	0.525898	{0.126118, 0.800752}	57.6637	ar	5710.	16011.2	{14770., 17264.}	180.407
15	R0	{1.62071, 5.98665}	{2.6992, 4.80907}	{{2.169, 2.21908}, {3., 12.7365}}	{66.544, 65.9186}	inf	12.	462.032	{155.836, 155.836}	3751.46	1.21713	newly	11.	509.23	{76., 633.}	4530.82	1.34319	Reff	0.547962	0.635625	{0.334387, 1.22283}	53.8155	ar	6635.	17602.9	{15990., 20776.}	165.303
16	R0	{1.67268, 7.7256}	{2.74107, 3.36482}	{{2.16061, 1.51578}, {3., 4.73764}}	{64.4204, 56.4458}	inf	38.	370.54	{153.499, 1140.72}	877.224	0.733628	newly	29.	763.2	{135., 3303.}	2533.45	1.0034	Reff	0.519367	0.654851	{0.11535, 2.1604}	77.7184	ar	6947.	16809.7	{14853., 25892.}	141.971
17	R0	{1.67805, 11.1417}	{2.65205, 2.50405}	{{2.08435, 0.726306}, {3., 4.64158}}	{58.0438, 77.5254}	inf	310.	212.534	{115.571, 454.976}	75.5789	0.428678	newly	183.	814.92	{103., 2221.}	383.137	0.352501	Reff	0.643364	0.569651	{0.168729, 1.3188}	67.5054	ar	6625.	16318.2	{14620., 22338.}	146.313
18	R0	{1.66743, 9.99046}	{2.00759, 4.68237}	{{0.507417, 0.189363}, {3., 11.4074}}	{75.5008, 63.7256}	inf	216.	193.453	{16.2889, 170.267}	61.738	0.347646	newly	133.	263.11	{9., 390.}	145.346	0.437069	Reff	0.574763	0.326217	{0.092243, 0.812033}	60.0751	ar	6899.	14879.3	{14328., 15338.}	115.673
19	R0	{1.70787, 9.85478}	{2.17095, 2.95029}	{{0.728257, 0.287377}, {3., 5.58799}}	{59.2199, 70.0624}	inf	121.	365.261	{15.5658, 148.969}	233.234	0.36562	newly	78.	435.9	{16., 363.}	486.564	0.561265	Reff	0.56428	0.369406	{0.112278, 0.5831}	42.7531	ar	6867.	14411.9	{13413., 16035.}	109.872
20	R0	{1.53809, 10.5848}	{2.62954, 2.40933}	{{1.36255, 0.486129}, {3., 5.08475}}	{75.2869, 77.2378}	inf	283.	801.678	{49.6692, 1563.78}	209.028	0.454487	newly	160.	1326.99	{65., 4247.}	748.381	0.721821	Reff	0.669684	0.487399	{0.0807177, 1.06689}	50.0438	ar	6108.	15466.5	{13464., 20672.}	153.216
21	R0	{1.59925, 6.74479}	{2.22322, 5.32644}	{{1.27219, 3.38854}, {3., 6.84907}}	{57.3306, 22.7605}	inf	59.	132.729	{55.7944, 201.2}	141.679	0.2055	newly	39.	220.16	{35., 264.}	472.564	0.326584	Reff	0.593482	0.475936	{0.236942, 0.775618}	38.6547	ar	6411.	15497.4	{14541., 19097.}	141.732
22	R0	{1.38779, 4.40977}	{2.72164, 3.90428}	{{2.13013, 1.31279}, {3., 12.1917}}	{96.1133, 68.2819}	inf	49.	587.777	{339.7, 996.459}	1099.54	0.955783	newly	54.	1262.99	{199., 3595.}	2238.87	1.12103	Reff	0.682793	0.741593	{0.42076, 1.5672}	45.3646	ar	5154.	17466.9	{15285., 22521.}	238.9
23	R0	{1.53657, 7.50352}	{2.45849, 4.66935}	{{1.49317, 0.293934}, {3., 7.45699}}	{61.9086, 37.7713}	inf	141.	398.099	{150.832, 381.446}	186.079	0.168495	newly	110.	463.07	{94., 425.}	328.064	0.309416	Reff	0.661647	0.464789	{0.162718, 1.05596}	52.5078	ar	5928.	15863.5	{14633., 18765.}	167.603
24	R0	{1.71131, 6.24526}	{2.74518, 3.74301}	{{2.25054, 1.53242}, {3., 4.97312}}	{60.4142, 40.0664}	inf	47.	229.202	{48.1449, 214.067}	394.402	0.517163	newly	36.	416.08	{54., 406.}	1058.78	0.696314	Reff	0.538036	0.53714	{0.164347, 0.652001}	52.9918	ar	6924.	15839.6	{14432., 20143.}	128.764
25	R0	{1.4412, 3.07729}	{2.5196, 6.61934}	{{0.764648, 0.0592822}, {3., 15.3138}}	{85.6744, 226.61}	inf	2.	411.741	{78.2898, 387.431}	20487.1	2.0921	newly	2.	1608.14	{88., 8674.}	80307.	2.23899	Reff	0.603863	0.723535	{0.122404, 2.23057}	93.3329	ar	5810.	18361.1	{15940., 24794.}	216.025
26	R0	{1.4159, 4.93858}	{2.39505, 2.96077}	{{1.57057, 2.58981}, {3., 3.28308}}	{69.154, 40.0481}	inf	48.	873.898	{62.9669, 1779.07}	1731.95	0.920523	newly	55.	1301.7	{100., 6020.}	2276.4	1.03976	Reff	0.683172	0.654218	{0.234198, 1.57119}	56.1219	ar	5255.	18038.5	{15236., 25259.}	243.264
27	R0	{1.57465, 7.05414}	{2.61258, 6.94512}	{{1.98047, 0.0761714}, {3., 16.0283}}	{65.915, 110.712}	inf	116.	294.754	{96.6787, 314.075}	168.265	0.370892	newly	88.	865.46	{64., 588.}	899.705	0.508863	Reff	0.599784	0.706872	{0.0546282, 1.98028}	67.6715	ar	6359.	17195.1	{15345., 23524.}	170.406
28	R0	{1.41422, 4.62402}	{2.21726, 5.0509}	{{1.22018, 1.78134}, {3., 8.62119}}	{68.8036, 69.9356}	inf	11.	187.619	{29.662, 498.477}	1612.62	1.02727	newly	15.	301.66	{23., 655.}	1919.87	0.980564	Reff	0.643046	0.702635	{0.229927, 1.22139}	51.8243	ar	5485.	16292.	{14779., 19930.}	197.028
29	R0	{1.41901, 3.63017}	{2.66536, 6.86821}	{{2.10051, 3.96246}, {3., 10.5664}}	{87.8322, 89.198}	inf	5.	570.093	{402.939, 798.348}	11301.9	2.03409	newly	11.	728.2	{207., 1054.}	6520.	1.65233	Reff	0.571577	0.591877	{0.180501, 0.884875}	58.9754	ar	5979.	17605.3	{16359., 18002.}	194.452
30	R0	{1.60719, 4.72984}	{1.91711, 6.44685}	{{0.0247164, 3.74959}, {3., 10.6656}}	{89.8996, 61.586}	inf	1.	538.56	{88.9487, 388.126}	53756.	2.40193	newly	3.	575.91	{88., 507.}	19097.	1.84701	Reff	0.566374	0.400812	{0.00296266, 0.775144}	70.141	ar	6479.	17218.2	{16104., 18700.}	165.754
31	R0	{1.38175, 6.26468}	{2.26798, 5.37599}	{{0.71165, 1.26857}, {3., 8.00331}}	{95.1394, 48.0026}	inf	50.	783.012	{158.023, 3344.23}	1466.02	0.679336	newly	41.	991.37	{95., 6190.}	2317.98	0.728118	Reff	0.648593	0.385003	{0.117402, 0.542858}	46.5164	ar	5397.	16052.3	{14334., 24052.}	197.43
32	R0	{1.4947, 8.8847}	{2.07582, 10.0394}	{{0.908947, 6.84558}, {3., 13.2865}}	{64.0461, 36.3264}	inf	106.	1582.54	{918.95, 918.95}	1393.94	1.00084	newly	69.	886.34	{397., 935.}	1186.43	0.959559	Reff	0.721193	0.406497	{0.171257, 0.781541}	50.3241	ar	5407.	17625.5	{15378., 21363.}	225.976
33	R0	{1.55891, 8.28978}	{1.90814, 4.13585}	{{0.190905, 2.67759}, {3., 5.45022}}	{90.377, 50.109}	inf	118.	281.318	{19.2348, 191.612}	164.837	0.36229	newly	80.	255.71	{19., 285.}	243.038	0.460541	Reff	0.628553	0.275314	{0.00132728, 0.598982}	57.8152	ar	6239.	16113.8	{15567., 16166.}	158.276
34	R0	{1.60764, 10.4729}	{2.22823, 3.88864}	{{0.827826, 3.21897}, {3., 5.82816}}	{65.7813, 62.8695}	inf	168.	929.455	{328.853, 2463.21}	453.247	0.48653	newly	102.	1062.06	{300., 3298.}	941.235	0.745195	Reff	0.600936	0.439712	{0.125342, 1.04987}	60.4396	ar	6495.	17004.6	{14993., 22209.}	161.81
35	R0	{1.48527, 5.74485}	{2.26464, 2.84665}	{{0.626797, 2.12784}, {3., 3.8593}}	{88.2637, 50.4486}	inf	59.	413.332	{41.6567, 1048.17}	612.146	0.65292	newly	58.	548.95	{51., 1660.}	854.569	0.741554	Reff	0.640597	0.421317	{0.128318, 0.703878}	44.1378	ar	5811.	16414.7	{15116., 21895.}	182.477
36	R0	{1.61457, 9.93364}	{2.79392, 5.98517}	{{2.28937, 0.0871566}, {3., 14.8144}}	{73.044, 78.8988}	inf	204.	660.821	{188.265, 703.2}	228.169	0.33445	newly	131.	1574.62	{149., 7476.}	1104.12	0.598449	Reff	0.638401	0.586817	{0.0748825, 0.769904}	59.2898	ar	6338.	17443.6	{15584., 24794.}	175.222
37	R0	{1.47831, 7.08139}	{2.59093, 2.96038}	{{2.09096, 1.07052}, {3., 5.04396}}	{75.2632, 58.1949}	inf	93.	292.392	{89.581, 433.019}	222.746	0.350166	newly	79.	788.72	{114., 1923.}	903.063	0.557381	Reff	0.683866	0.572021	{0.118356, 1.27768}	61.1465	ar	5530.	16714.6	{15078., 22794.}	202.254
38	R0	{1.73445, 9.8858}	{2.57941, 2.68968}	{{2.06536, 1.11991}, {3., 4.94944}}	{48.7163, 72.7925}	inf	168.	178.251	{107.561, 245.931}	69.9754	0.247424	newly	85.	603.37	{101., 749.}	614.788	0.448528	Reff	0.583642	0.662564	{0.313869, 1.47606}	57.6689	ar	6842.	16159.8	{14568., 19563.}	136.185
39	R0	{1.66621, 11.4824}	{2.64247, 5.37563}	{{2.0985, 3.96461}, {3., 6.31631}}	{58.5918, 53.1837}	inf	221.	178.128	{88.5559, 297.416}	54.1531	0.291278	newly	130.	218.02	{66., 377.}	116.308	0.333472	Reff	0.657653	0.602934	{0.337952, 0.752088}	41.4047	ar	6473.	15707.5	{14387., 17669.}	142.662
40	R0	{1.60694, 9.95741}	{2.65126, 2.34447}	{{1.96561, 0.682142}, {3., 5.47658}}	{64.9884, 76.9667}	inf	179.	443.58	{177.574, 780.121}	157.674	0.19204	newly	116.	988.78	{191., 2812.}	758.155	0.646034	Reff	0.592157	0.701342	{0.252387, 1.96541}	60.0741	ar	6625.	17418.2	{14777., 23201.}	162.916
41	R0	{1.55128, 4.71911}	{2.49865, 6.15071}	{{1.54049, 3.68044}, {3., 10.7384}}	{61.3339, 58.9491}	inf	16.	869.974	{48.0863, 497.36}	5338.86	1.28139	newly	16.	1148.5	{51., 3310.}	7079.5	1.32395	Reff	0.605309	0.746793	{0.0769595, 2.36384}	81.1427	ar	6141.	18842.5	{15933., 25840.}	206.831
42	R0	{1.60063, 6.81118}	{2.38274, 7.60455}	{{1.32909, 0.811494}, {3., 18.9452}}	{61.2945, 73.7924}	inf	61.	795.74	{80.6456, 949.557}	1208.58	0.886636	newly	44.	1023.41	{26., 1094.}	2239.07	1.00777	Reff	0.565983	0.432058	{0.177728, 0.716632}	51.698	ar	6528.	17554.9	{15497., 23494.}	168.917
43	R0	{1.56986, 5.44652}	{2.57681, 5.17229}	{{1.85624, 0.44992}, {3., 15.6452}}	{64.1426, 120.956}	inf	36.	651.831	{107.131, 321.745}	1710.64	0.931936	newly	40.	1273.93	{56., 7150.}	3085.43	1.03292	Reff	0.586343	0.679439	{0.371844, 1.05416}	39.7933	ar	6312.	17769.	{15762., 23396.}	181.512
44	R0	{1.47523, 8.07785}	{2.84266, 9.24822}	{{2.34443, 6.67771}, {3., 11.5532}}	{92.6929, 30.7817}	inf	154.	793.439	{133.245, 1979.44}	419.903	0.485741	newly	118.	613.43	{68., 1791.}	432.415	0.459862	Reff	0.674918	0.523551	{0.0962438, 0.837197}	51.3497	ar	5692.	17636.4	{15611., 24438.}	209.845
45	R0	{1.6417, 7.69429}	{2.69374, 6.71271}	{{2.07195, 3.99302}, {3., 11.5477}}	{64.0825, 48.8161}	inf	70.	255.975	{192.915, 296.677}	266.02	0.540384	newly	45.	342.44	{114., 443.}	660.978	0.732719	Reff	0.547999	0.646473	{0.165316, 1.67845}	63.58	ar	6735.	15762.7	{14648., 18707.}	134.041
46	R0	{1.38865, 5.83473}	{2.49867, 4.02746}	{{1.56491, 2.50195}, {3., 7.00717}}	{79.9353, 41.5926}	inf	81.	1077.57	{322.667, 3099.58}	1230.79	0.907794	newly	88.	1461.2	{283., 4029.}	1561.	0.927983	Reff	0.6778	0.509926	{0.143437, 0.895029}	51.2917	ar	5233.	18183.6	{15606., 26152.}	247.479
47	R0	{1.45289, 6.87903}	{2.56126, 3.20477}	{{2.10462, 0.374508}, {3., 4.71897}}	{76.2874, 53.4124}	inf	113.	365.878	{170.649, 857.526}	232.83	0.357365	newly	105.	1061.67	{148., 1281.}	917.476	0.516928	Reff	0.690704	0.628716	{0.228323, 1.54062}	62.2387	ar	5379.	16651.9	{14194., 21606.}	209.572
48	R0	{1.69416, 9.4831}	{2.79929, 10.2342}	{{2.33096, 2.82786}, {3., 18.286}}	{65.2317, 80.8791}	inf	191.	1104.16	{152.687, 3370.7}	490.991	0.609805	newly	108.	1098.28	{71., 1891.}	929.852	0.680853	Reff	0.561783	0.602289	{0.224427, 1.31253}	65.3328	ar	6925.	18055.2	{15277., 25199.}	160.725
49	R0	{1.47843, 7.16286}	{2.39142, 8.0384}	{{1.51124, 2.11339}, {3., 10.934}}	{61.7543, 57.0968}	inf	84.	238.911	{7.78443, 265.374}	211.483	0.327752	newly	57.	307.27	{4., 197.}	472.684	0.43555	Reff	0.694419	0.521258	{0.159211, 0.941468}	53.5791	ar	5449.	14865.4	{13720., 20117.}	172.809
50	R0	{1.68843, 5.72427}	{2.66528, 8.83547}	{{2.00079, 3.05245}, {3., 15.9099}}	{57.8553, 97.7492}	inf	27.	380.412	{10.2988, 1135.45}	1334.98	0.821274	newly	30.	540.12	{8., 862.}	1738.27	0.735021	Reff	0.541311	0.507801	{0.0628392, 1.19385}	60.5356	ar	6800.	17445.8	{15725., 23971.}	156.556
